# Assessment of *Giardia* and *Cryptosporidium* Assemblages/Species and Their Viability in Potable Tap Water in Beni-Suef, Egypt Using Nested PCR/RFLP and Staining

**Published:** 2019

**Authors:** Doaa HAMDY, Ayman El-BADRY, Wegdan ABD EL WAHAB

**Affiliations:** 1. Department of Medical Parasitology, College of Medicine, Beni-Suef University, Beni-Suef, Egypt; 2. Department of Microbiology-Medical Parasitology Section, College of Medicine, Imam Abdulrahman Bin Faisal University, Dammam, Saudi Arabia

**Keywords:** *Cryptosporidium* spp., Tap water, Egypt, *Giardia*

## Abstract

**Background:**

The protozoan *Giardia* and *Cryptosporidium* are responsible for most water-borne diseases all over the world. The extent and number of outbreaks of waterborne diseases suggests a significant risk of their potential transmission via drinking water. This study aimed to document the prevalence and viability of *Giardia* and *Cryptosporidium* (oo) cysts in tap water samples in Beni-Suef Governorate, Egypt and to detect the predominant *Giardia* and *Cryptosporidium* assemblages/species using nested PCR/ Restriction Fragment Length Polymorphism (RFLP) confirmed by further sequencing of positive samples.

**Methods:**

A total of 80 tap water samples were collected throughout a year from four big centers and filtered using the membrane filtration method. Samples were stained by Lugol's iodine, Modified Zeihl-Neelsen (MZN) (to detect prevalence) and trypan blue stain (to detect viability). Nested PCR-RFLP and sequencing were used for molecular characterizations and genotyping of the detected *Giardia* and *Cryptosporidium*.

**Results:**

*Giardia* and *Cryptosporidium* DNA was detected in 20 (25%) and 29 (36.3%) samples respectively, with predominance of *Giardia* assemblage B (85%) and *C. hominis* (75.9%). The prevalence and viability of both parasites (oo) cysts showed seasonality which peaked in summer and were greater in Beba center and in rural areas.

**Conclusion:**

To our knowledge, no studies have been done in these areas before. The anthroponotic transmission has an important role in giardiasis and cryptosporidiosis epidemiology in this studied area.

## Introduction

Water plays an important role in the transmission of many different pathogenic microorganisms such as bacteria, viruses, fungi, protozoa, and helminthes. Water-borne pathogenic protozoa including *Cryptosporidium* spp*., Cystoisospora belli, Cyclospora cayetanensis, Microsporidia, Giardia lamblia, Entamoeba histolytica* and free-living amoebae are responsible for emerging cases of waterborne diseases (1).

Both *Giardia* and *Cryptosporidium* are the most common waterborne and foodborne parasites all over the world (2). They are transmitted by sustained anthroponotic and zoonotic cycles including many species and genotypes (3, 4). The robust form ((oo) cyst), is resistant to common disinfectants at the exposure times and the concentrations usually applied in water treatment processes. The infectious doses of both parasites are as low as 10 cysts (3) and 30 oocysts (5) for *Giardia* and *Cryptosporidium*, respectively. This problem is potentiated by the wide range of infected hosts shedding large number of infective (oo) cysts causing environmental contamination, particularly in water sources (4).

Outbreaks of water borne diseases showed a great increase not only in number but also in extent (4). These outbreaks are attributed to contamination of water sources by soil or dead animal's thrown into them, agricultural runoffs, snowmelts, biosolids and heavy rainfall (6, 7). Exposure of uncovered water tanks to excreta of infected rodents and birds and inadequate treatment of drinking water may be additional factors (8, 9).

The *Cryptosporidium* genus is comprised of 30 species and more than 40 genotypes (10). Twelve species were reported to infect mammals, of which *C. hominis* and *C. parvum* account for over 90% of human infections (11).

*G. lamblia* is a parasite of mammals as well as humans. Six species are reported in the *Giardia* genera according to its morphological characteristics and infected hosts (12). It is generally accepted that *G. lamblia* is a complex of eight distinct genetic groups (designated A–H). These groups are identical in morphology but differ in genomic mutations (13). Genetic groups A and B which are subdivided into five sub-groups (named AI–III and BIII–BIV), mainly infect humans (14).

In Egypt, waterborne diseases represent a major public health problem. The Nile River, the main source of drinking water in Egypt, is polluted by human activities, reservoir animal hosts, sewage and industrial discharge, and run-off from agricultural fields. The problem is augmented in some rural Egyptian villages which obtain their water supply from unprotected streams and ground water (15).

This problem highlights the need to determine prevailing protozoa species and genotypes contaminating water sources to evaluate the risks on human and animal health, and outline proper control measures. However, some of the protozoa (oo) cysts contaminating water are non-viable and have no threat to the public health. Consequently, there is a great interest in developing in-vitro techniques capable of determining (oo) cyst viability (16).

## Materials and Methods

### Study design and water samples collection

The present work is a descriptive analytical study conducted in Beni-Suef Governorate, Egypt. A total of 80 tap water samples (10 L/sample) were collected in sterile containers over one year, from April 2016 to April 2017. Samples were collected from four different big centers representing different communities in Beni-Suef Governorate, namely Beni-Suef, Naser, El Wasta and Beba centers. From each center, 20 tap water samples were collected (10 each from urban and rural areas). Date and place were labeled on the containers. Distribution of the collected 80 samples in different seasons was as follows: 17, 21, 12 and 30 samples in spring, summer, autumn and winter seasons, respectively.

### Water samples filtration and processing

Water samples were transferred immediately after collection to the laboratory of the Medical Parasitology Department, Faculty of Medicine, Beni-Suef University, stored at 4°C until processed on the same day. Each water sample was filtered using a stainless steel filtration unit with an oil free pump according to the manufacturer's instructions through 47-mm diameter sterile nylon membrane filter with 1 μm pore size. The membrane filter was folded twice lengthwise with the upper surface facing out, soaked in phosphate buffered saline (PBS) in a 15-ml conical centrifuge tubes for two hours and then centrifuged at 6000 rpm for 10 min. (17). The supernatant was decanted. Part of the sediment was examined parasitologically and another part was kept at −20°C for further molecular assays.

### Parasitological examination

A drop of the pellet was put on a slide and examined using saline and iodine wet mount smears using 40X objective lens for detection of *Giardia* cysts (17, 18). Part of the pellet was preserved in 10% buffered formalin solution, stained by MZN and examined by 40X and 100X objectives to detect *Cryptosporidium* (oo) cysts (9,17).

Trypan Blue vital stain (Euromedex, France) was used for detecting the viability of (oo) cysts in fresh positive water samples following the manufacturer instructions (9, 17, 19).

### Molecular assays

Genomic DNA was extracted from fresh frozen pellets using Stool DNA Mini Kit of FavorPrep (Favorgen Biotech corporation ping-Tung, 908 Taiwan) following the instructions of the manufacturer. The concentration and purity of extracted DNA was determined.

Extracted DNA was amplified by nested PCR-RFLP for detection and typing of *Cryptosporodium* and *Giardia*. Nested PCR targeting *Cryptosporidium* oocyst wall protein (COWP) gene was done as reported earlier (20, 21). Another nested PCR targeting *Giardia* beta (β) giardin gene was performed (22, 23).

Positive *Cryptosporidium* and *Giardia* DNA produced by nested PCRs were cleaved by RsaI restrictive enzyme (Fermentas UAB, V. Graiciuno 8, LT-02241 Vilnius, Lithuania) and HaeIII restrictive enzyme (Fermentas UAB, V. Graiciuno 8, and LT-02241 Vilnius, Lithuania), respectively. Digested nested PCR fragments were visualized by 3.2% agarose gels electrophoresis to determine the *G. lamblia* assemblages and *Cryptosporidium* genotypes.

Species/assemblage identification of all positive PCR-products was purified by PCR purification kit. Purified PCR products were bidirectionally sequenced using the big Dye Terminator version 3.1 cycle sequencing kit (Applied Biosystems) and the nested PCR primers for each parasite on an ABI 310 sequencer (Applied Biosystems) according to the manufacturer's instructions. The obtained sequences were compared to the GenBank reference sequences using nucleotide BLAST search at NCBI website (http://www.ncbi.nlm.nih.gov) to determine the *Cryptosporidium* and *Giardia* species/assemblages. Sequencing of PCR products and phylogenetic analysis were aligned by the BioEdit alignment program (24)

### Statistical analysis

Results were displayed in tables and analyzed statistically using SPSS-23 (IBM, Somers, NY, USA) software. Descriptive data were expressed as numbers and percentages. Differences in discrete variables were compared and assessed for significance by Chi square-test. Diagnostic yield (accuracy, sensitivity, specificity, positive predictive value (PPV), negative predictive value (NPV) and kappa agreement) of conventional microscopy and staining results was measured compared to nested PCR results as a reference standard. The final model included all variables with significant *P* at <0.05.

### Ethical consideration

The protocol of this study was approved by Beni-Suef University, Scientific Research Development Unit, Projects Funding and Granting Unit. This article does not contain any studies with human or animal subjects. Individuals in contaminated areas were informed about the obtained results of the research for subsequent precautions.

## Results

Out of the 80 collected tap water samples, *Giardia* cysts were detected in 6 (7.5%) samples by lugol's iodine while *Cryptosporidium* oo-cysts were detected only by MZN stain in 13 (16.3%) samples. According to nested PCR results, *Cryptosporidium* and *Giardia* (oo) cysts were identified in 29 (36.3%) and 20 (25%) samples, respectively with statistical significance (*P* <0.001) (Table 1). Eleven samples (13.7%) had mixed infection of both parasites by nested PCR. Diagnostic yields of microscopic examination of water samples by iodine and MZN in relation to nested PCR are presented in (Table 2).

**Table 1:** *Giardia* and *Cryptosporidium* (oo) cysts detection in examined water samples by microscopy (lugol's iodine and MZN stain) and nested PCR

***Diagnostic technique***			***Nested PCR***			***P ***
			***Positive n. (%)***	***Negative n. (%)***	***Total n. (%)***	
Lugol's Iodine	*Giardia* cysts	Positive	6 (7.5)	0 (0.0)	6 (7.5)	<0.001 ^*^
		Negative	14 (17.5)	60 (75)	74 (92.5)	
		Total	20 (25)	60 (75)	80 (100)	
MZN	*Cryptosporidium* oocysts	Positive	13 (16.3)	0 (6.2)	13 (16.3)	<0.001 ^*^
		Negative	16 (20)	51 (63.8)	67 (83.8)	
		Total	29 (36.3)	51 (63.8)	80 (100)	

* Significant (*P* < 0.05)

**Table 2:** Diagnostic yield of iodine and MZN stained smears to detect *Giardia* and *Cryptosporidium* (oo) cysts in water samples considering nested PCR as a reference test

***Variable***	***Iodine stained smears Giardia cyst (%)***	***MZN stain Cryptosporidium oocyst (%)***
Sensitivity	30	44.8
Specificity	100	100
PPV	100	100
NPV	81.1	76.1
Accuracy	82. 5	80

RFLP results revealed the predominance of *Giardia* assemblage B (85%) and *Cryptosporidium hominis* (75.9%) with high statistical significance (*P*<0.001) (Table 3).

**Table 3:** *Giardia* assemblages and *Cryptosporidium* species identified in tested water samples using nested PCR-RFLP

***Protozoa***		***Nested PCR-RFLP n. (%)***	***P***
*Giardia* assemblages	Assemblage A	3 (15)	<0.001 ^*^
	Assemblage B	17 (85)	
	Total	20 (100)	
*Cryptosporidium* genotypes	*C. hominis*	22 (75.9)	<0.001 ^*^
	*C. parvum*	6 (20.7)	
	*C. hominis + C. parvum*	1 (3.4)	
	Total	29 (100)	

* Significant (*P* < 0.05)

**Fig. 1:**
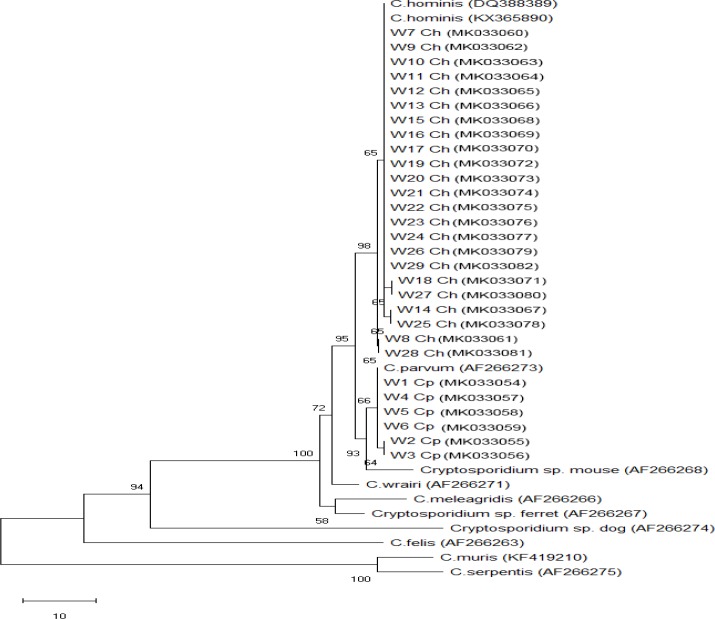
Phylogenetic tree of COWP sequences of *Cryptosporidium* from water samples and reference sequences from the GenBank. Neighborjoining tree showing the evolutionary history of *Cryptosporidium* isolates, inferred by distance-based analysis of *Cryptosporidium* COWP gene sequence. Bootstrap value is 500 with the sum of the branch length =0.1. The monophyletic clades of *Cryptosporidium parvum* (samples W1–W6) and *Cryptosporidium hominis* (samples W7–W29) were supported by high bootstrap values

**Fig. 2:**
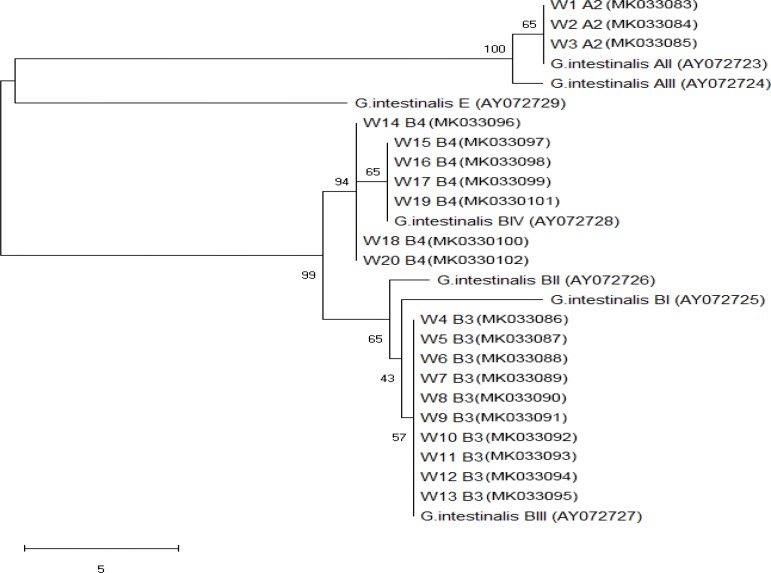
Phylogenetic tree of Beta giardin sequences of *Giardia intestinalis* from water samples and reference sequences from the GenBank. Neighborjoining tree showing the evolutionary history of *Giardia* isolates, inferred by distance-based analysis of *Giardia intestinalis* Beta giardin gene sequence. Bootstrap value is 500 with the sum of the branch length =0.1. The monophyletic clades of *Giardia intestinalis* AII (samples W1–W3), *Giardia intestinalis* BIII (samples W4–W13) and *Giardia intestinalis* BIV (samples W14–W20) were supported by high bootstrap values

Phylogenetic analyses of the SSU rDNA showed that analysis of *Giardia* assemblages revealed that all three assemblage A samples by nested PCR were of AII, while 17 assemblage B samples were of BIII (10 samples.) and BIV (7 samples) representing 58.8% and 41.2%, respectively. Subgenotyping of *Cryptosporidium* species confirmed detection of *C. parvum* and *C. hominis*. All species were matched with the same cluster of *Giardia/Cryptosporidium* subtypes in the NCBI database with no genetic variability. *Cryptosporidium/Giardia* isolates sequence data were placed in GenBank with accession numbers (MK033054-MK033102) (Figs. 1, 2).

Beba center had the highest positive rate of *Giardia* and *Cryptosporidium* water contamination (40% and 37.9% respectively), while El Wasta center had the lowest rate (15% and 13.8 %) without statistical significance. Water contamination was higher in rural areas (75% for *Giardia* and 58.6% for *Cryptosporidium*) than urban areas. These data showed statistical significance for *Giardia* only (*P* =0.009) (Table 4).

**Table 4:** Distribution of *Giardia* and *Cryptosporidium* using nPCR according to geographic area and represented communities

***Variables***	***Giardia (nested PCR)***		***P***	***Cryptosporidium (nested PCR)***		***P***
	***Positive n. (%)***	***Negative n. (%)***		***Positive n. (%)***	***Negative n. (%)***	
Geographic area (20 samples/center)						
Beni-Suef	4 (20)	16 (26.7)	0.2	7 (24.1)	13 (25.5)	0.1
El Wasta	3 (15)	17 (28.3)		4 (13.8)	16 (31.4)	
Nasser	5 (25)	15 (25)		7 (24.1)	13 (25.5)	
Beba	8 (40)	12 (20)		11(37.9)	9 (17.6)	
Representing community (40/point)						
Urban	5 (25)	35 (58.3)	0.009^*^	12 (41.4)	28 (54.9)	0.1
Rural	15 (75)	25 (41.7)		17 (58.6)	23 (45.1)	
Total	20 (100)	60 (100)		29 (100)	51 (100)	

* Significant (*P* < 0.05)

**Fig. 3:**
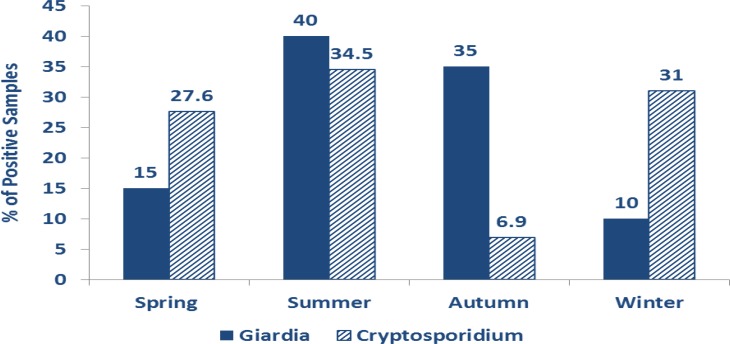
Seasonal distribution of positive *Giardia* cysts (*P* =0.002) and *Cryptosporidium* oocyst (*P* =0.2)

Both parasites were detected in all seasons with a seasonal fluctuation and peak in summer (40% for *Giardia* and 34.5% for *Cryptosporidium*). The lowest rate of *Giardia* cysts was in winter and the lowest rate of *Cryptosporidium* oocysts was in the autumn season with statistical significance regarding *Giardia* only (Fig. 3).

**Fig. 4:**
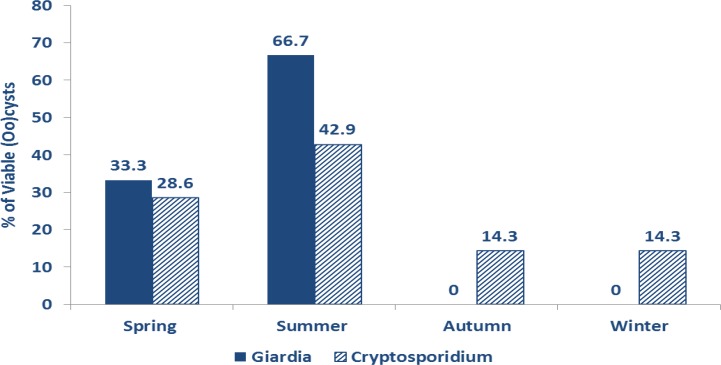
Seasonal distribution of viable *Giardia* cysts (*P* =0.004) and *Cryptosporidium* oocyst (*P* =0.4)

*Cryptosporidium* and *Giardia* (oo) cysts were viable in 24.1% and 15% respectively, without statistical significance between sampled areas in different centers. Viability was higher in rural areas and in summer season with statistical-significance with *Giardia* only (Table 5 and Fig. 4).

**Table 5:** Detection of viability of *Giardia* cysts and *Cryptosporidium* oocysts using trypan blue stain according to different geographic areas and represented communities

***Variables***	***Giardia using nPCR (n.=20)***		***P***	***Cryptosporidium using nPCR (n.=29)***		***P***
	***Viable No. (%)***	***Non-viable No. (%)***		***Viable No. (%)***	***Non-viable No. (%)***	
Geographic area (20 samples/center)						
Beni-Suef	0 (0)	4 (23.5)	0.5	1 (14.3)	6 (27.3)	0.4
El Wasta	1 (33.3)	2 (11.8)		1 (14.3)	3 (13.6)	
Nasser	1 (33.3)	4 (23.5)		2 (28.6)	5 (22.7)	
Beba	1(33.3)	7 (41.2)		3 (42.9)	8 (36.4)	
Representing community (40/point)						
Urban	1 (33.3)	4 (23.5)	0.03^*^	3 (42.9)	9 (40.9)	0.5
Rural	2 (66.7)	13 (76.5)		4 (57.1)	13 (59.1)	
Total	3 (15)	17 (85)		7 (24.1)	22 (75.9)	

* Significant (*P* < 0.05)

## Discussion

Water is considered the main environmental route for transmitting *Giardia* cysts and *Cryptosporidium* oocysts (25). In Egypt, surface water is the main drinking water source with an absence of mandatory programs for monitoring pathogenic protozoan parasites in water.

Prevalence of *Giardia* and *Cryptosporidium* showed varied results in Egypt. In Alexandria Governorate, *Giardia* and *Cryptosporidium* (oo) cysts were identified in 36.7% and 100%, respectively in tank water samples (9). In Giza Governorate, *Giardia* cysts were detected in 50% and 33% of tap water in Abo-El Nomros and El Hawamdia, respectively (26). In Assuit Governorate, *Cryptosporidium* oocysts were detected in 50% of drinking water samples (27) and 79% of drinking water supply of Assiut university hospitals (28). Recent study at Fayoum Governorate had detected 52.6%, 13.7% of *Cryptosporidium* spp. and *G. lamblia*, respectively in tap water and storage water tanks (18). In El-Minia Governorate, *Giardia* and *Cryptosporidium* were detected in 0% and 12.5%, respectively in tap water (29). In Gharbiya Governorate, *Giardia* and *Cryptosporidium* were detected in 13% and 7.4%, respectively in tap water samples (17).

Similar results were reported worldwide. *Cryptosporidium* was detected in 51% and 25%, while *G. lamblia* was detected in 0.62% and 2.4% of tap water samples in Jeddah and Makkah, respectively (30). On the other hand, in Iran Feiz Hadad *et al.* detected 0% of both parasites in filter system household tap water samples (31).

In Spain, both parasites were detected in 26.8% of examined water samples (32). In the UK Nichols *et al.* detected *Cryptosporidium* in 100% of drinking water samples using PCR (33). Hashimoto *et al.* found *Giardia and Cryptosporidium* in 12% and 35%, respectively of filtered water samples from a water plant in Japan (34). Lower detection rates were reported in the north of Portugal, *Giardia* was detected in 8.4% and *Cryptosporidium* was detected in 10.2% in drinking water samples (25).

In our study, there was seasonality of prevalence and viability of both *Giardia* and *Cryptosporidium* (oo) cysts which were higher in summer season. Seasonality of *Cryptosporidium* and *Giardia* prevalence in water had been recorded worldwide (35) and in Egypt (17, 28, 29) and was confirmed by seasonality of human infection (36, 37).

Peaking of *Cryptosporidium* and *Giardia* (oo) cysts prevalence in water in summer may be attributed to warm temperatures, humidity and stagnation of water that could increase incidence of parasites, prolong the infective period and the transmission of (oo) cysts, and promote more cyst contact with populations (38). Possibly, the key determinant of distinct seasonality is the increase of human outdoor activity during the summer season, which fosters more transmission of *Cryptosporidium/Giardia* (oo) cysts (39).

Our study showed seasonal variation in viability of detected *Giardia* cysts (15%) and *Cryptosporidium* oocysts (24.1%). This obtained viability may be attributed to their ability to persist in the environment and resist the conventional disinfection process and chlorination practices generally applied in drinking water treatment (32, 15).

The fact that flow cytometry accurately estimates the viability and parasite load in water samples with more sensitivity than trypan blue stain (9,17), may mean that the obtained viability percentage in our study is perhaps lower than what we expected.

Predominance of anthroponotic *Cryptosporidium* species (*C. hominis*) and *Giardia* assemblage B was reported in both urban and rural areas which suggest that human activities with person-to-person transmission rather than zoonotic transmission are the main source of water contamination.

Even in humans, there are nearly 12 species/genotype of *Cryptosporidium* have been reported, though *C. hominis*, *C. parvum*, *C. ubiquitum*, and *C. meleagridis* are the most common causative agents (11). In the case of giardiasis, although A and B are the main etiologies for human infections (14), it is worth noting that humans infections by assemblages C, D, E, and F have been sporadically reported particularly in immunocompromised patients and children (40).

In the present study, microscopy of MZN stained smear improved *Cryposporidium* oocyst detection by 13.6%. Although it stills a method of limited sensitivity (44.8 %) compared to nested PCR results, it was used in our study due to its safety, accuracy and simplicity than other stains for identifying *Cryptosporidium* species in water samples as confirmed by previous studies (9,17,28, 41).

In the present study, *Giardia* and *Cryptosporidium* (oo) cysts were detected at higher prevalence rate in rural areas than urban area. This may be attributed to the fact that rural populations in Egypt obtain their water supply from unprotected streams and ground water. In addition, there is an increase in water contamination due to the lack of proper infrastructure and inefficient water treatment procedures in rural areas (42). To our knowledge no studies have been done in these areas before.

## Conclusion

Detection of the seasonal prevalence of parasites in drinking water system aids to establish efficient control measures that should be applied in high-risk seasons to reduce the rate of infection. The obtained results highlighted the compromised water sanitation in Beni-Suef Governorate, Egypt and the need for proper control measures and effective water sanitation programs. The predominance of *Cryptosporidium* and *Giardia* species/assemblages emphasizes that anthroponotic transmission has an important role in cryptosporidiosis and giardiasis epidemiology in this studied area.
